# Effects of extracorporeal circulation with different time on platelet count after cardiac surgery: a retrospective study based on medical records

**DOI:** 10.1038/s41598-023-43334-0

**Published:** 2023-09-26

**Authors:** Na Wang, Ting Zhao, Jiabei Li, Sisi Zeng, Jixiang Wan, Xuechao Li, Fangjun Wang

**Affiliations:** Department of Anesthesiology, Affiliated Hospital of North Sichuan Medical College, Nanchong, Sichuan China

**Keywords:** Cardiology, Medical research

## Abstract

Our objective was to observe the effects of extracorporeal circulation (ECC) with different time on platelet count in patients undergoing cardiac surgery. A total of 427 patients who underwent elective cardiac surgery under ECC in affiliated hospital of north Sichuan medical college from January 1, 2018 to July 31, 2021 were divided into three groups according to ECC time. We concluded that thrombocytopenia was common after ECC, maximum drop of the platelet counts after ECC was usually seen on the second day after ECC, and platelet counts started to recover on the fifth day after ECC. With the extension of ECC time, the drop in platelet counts is more pronounced, the volume of perioperative blood loss and blood products transfusion are more, and the recovery level and speed of platelet counts is lower.

Thrombocytopenia often occurred after cardiac surgery under ECC, resulting in acute renal injury, postoperative infection, prolonging the hospitalization time postoperatively, and even increasing the postoperative mortality of patients^1^. Platelet count after cardiac surgery under ECC was lower than that without ECC, and the risk of postoperative bleeding was also higher^2^. The study suggested that ECC was a major risk factor leading to thrombocytopenia after operation^1^. However, there are few studies on the effects of ECC time on platelet count in patients after cardiac surgery, especially the effects of different ECC time on postoperative platelet count has not been reported. The purpose of this study was to conduct a retrospective study to observe the effects of ECC with different time on platelet count in patients undergoing cardiac surgery, so as to provide reference for improving the prognosis of patients undergoing cardiac surgery.

## Study design

This was a retrospective, single-center study (Clinical trials. gov ChiCTR2100053748, November 28, 2021), approved by Medical Ethics Committee of Affiliated Hospital of North Sichuan Medical College (NO. 2021ER148-1). Informed consent was obtained from all subjects. Inclusion criteria: from January 2018 to July 2021, adult patients (aged ≥ 18 years) who underwent ECC cardiac surgery at the Affiliated Hospital of North Sichuan Medical College; the platelet count before operation was (100–300) × 10^9^/L; ASA grade was II or III. Exclusion criteria: incomplete data; perioperative platelet transfusion; antiplatelet drugs and/or thrombopoietic drugs were used before operation (such as received antibiotics within 2 weeks prior to surgery, carbamazepine, naproxen, ibuprofen, amiodarone, ranitidine, and haloperidol), developed an infection within 48 h of surgery, unclear documentation of perioperative antimicrobial prophylaxis, end-stage renal disease, procedures performed for infective endocarditis. According to the above inclusion and exclusion criteria, a total of 427 patients were included in this study, and divided into ≤ 120 min group (group A), 120–180 min group (group B) and > 180 min group (group C) according to ECC time, with 140,149,138 cases in group A, B and C respectively. Primary outcome was the platelet count before operation (T_1_), at the end of operation (T_2_), on the first day (T_3_), the second day (T_4_) and the fifth day after operation (T_5_). Secondary outcomes included the variation of MPV and absolute monocyte count at T_1_, T_2_, T_3_, T_4_ and T_5_; general information: gender, age, weight, type of operation and ASA grade; the volume of intraoperative blood loss, the amount and type of blood products transfused and the dose of heparin and protamine.

### Statistical analysis

Statistical analysis was performed by using SPSS 23.0 statistical software. Categorical variables were presented as frequencies and percentages, whereas continuous variables with normal distribution were expressed as mean ± standard deviation and median for abnormal distribution data. Comparison among groups was performed by one-way ANOVA with a post hoc analysis, comparison at different time points was performed by repetitive measurement and analysis of variance with a Bonferroni correction. Categorical data were analyzed by Pearson’s *X*^*2*^ test or Fisher’s exact test. *P-*value < 0.05 was considered statistically significant.

## Results

**Figure 1:**
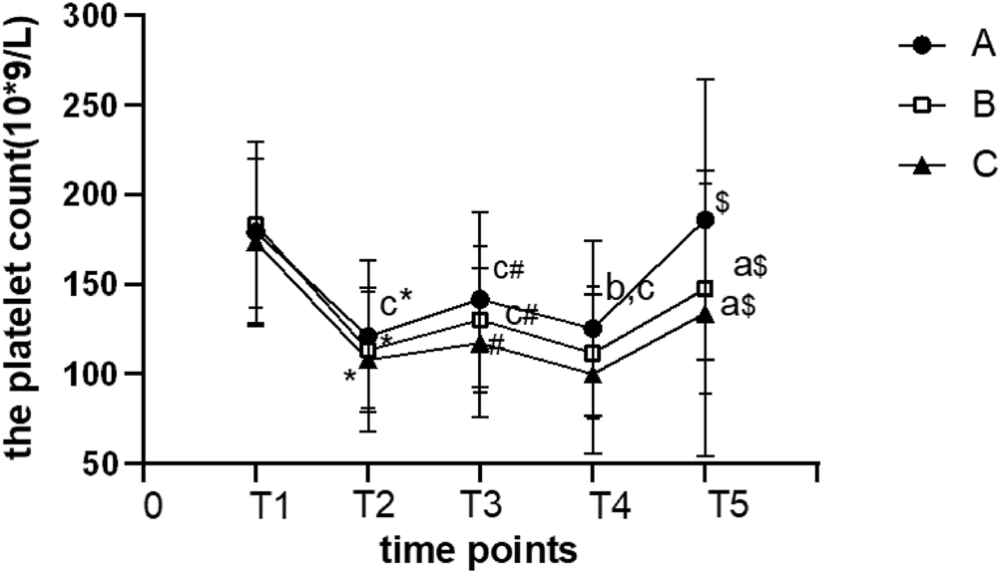
The platelet count at different time points in all groups. *p < 0.05 vs. T1; ^#^*p* < 0.05 vs. T2; ^$^*p* < 0.05 vs. T4. ^a^*p* < 0.05 vs. group A, ^b^*p* < 0.05vs. group B; ^c^*p* < 0.05vs. group C.

There was no significant difference in platelet count at T_1_ among the three groups (*p* > 0.05). Platelet count in all groups was reduced significantly at T_2_ compared with T_1_ (*p* < 0.05). Compared with T_2_, platelet count in each group increased slightly at T_3_ (*p* < 0.05) and decreased to the T_2_ level at T_4_ (*p* > 0.05). Platelet count was lower within 2 days after operation (*p* < 0.05) and increased significantly at T_5_ when compared with T_1_ in all groups (*p* < 0.05). Platelet count in groups B and C was significantly lower at T_5_ than at T_1_ (*p* < 0.05). Platelet count at T_2_ was significantly lower in group C than in group A (*p* < 0.05); Platelet count at T_3_ was significantly lower in group C than in groups A and B (*p* < 0.05); Platelet count at T_4_ in groups B and C was significantly lower than that in group A (*p* < 0.05); The recovery level of platelet count at T_5_ was significantly higher in group A than in groups B and C (*p* < 0.05) (Fig. 1). There were weak inverse correlations between platelet count and ECC time at T_2_ (r =  − 0.133, *p* < 0.05), T3 (r =  − 0.210, *p* < 0.05), T4 (r =  − 0.218, *p* < 0.05) and T_5_(r =  − 0.228, *p* < 0.05).

**Figure 2:**
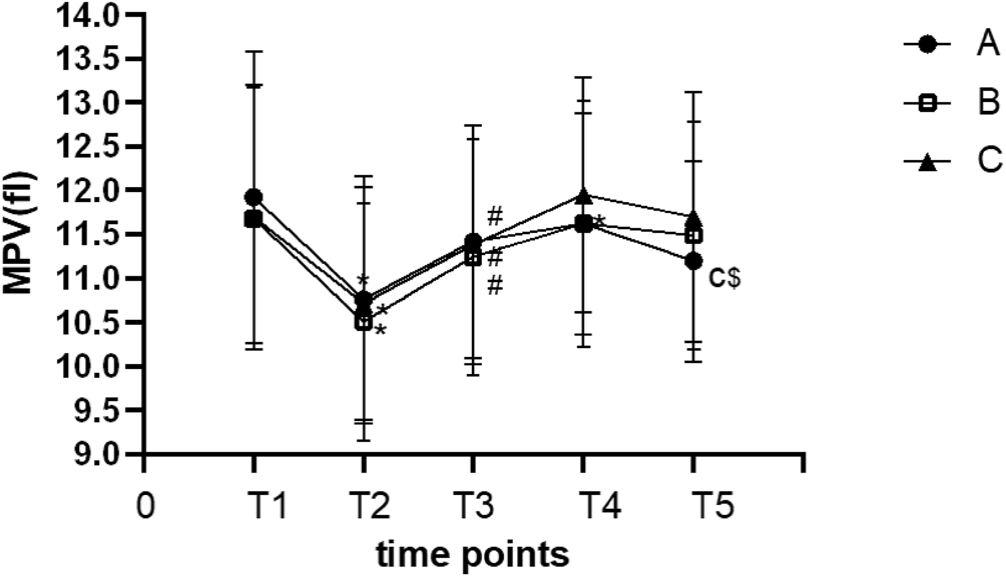
The MPV at different time points in all groups. *p < 0.05 vs. T1; ^#^*p* < 0.05vs. T2; ^$^*p* < 0.05vs. T4. ^c^*p* < 0.05 vs. group C.

**Figure 3:**
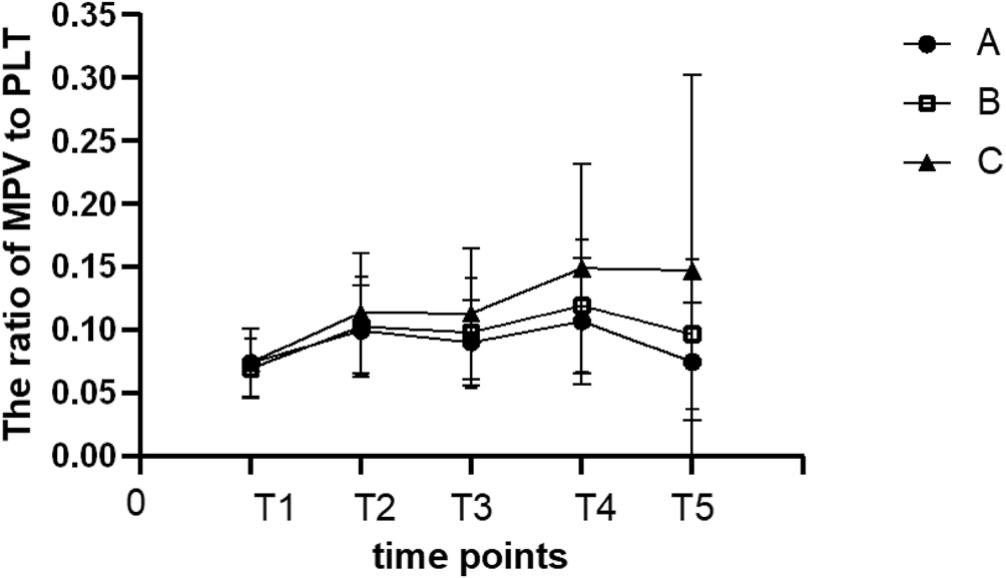
The ratio of MPV to platelet count at different time points in all groups.

The MPV in each group was significantly lower at T_2_ than at T_1_ (*p* < 0.05); The MPV at T_3_increased significantly compared with T_2_ (*p* < 0.05). The MPV in group A was lower at T_4_than at T_1_ (*p* < 0.05) and lower at T_5_than at T_4_. There was no difference in the MPV among the three groups at T_1_, T_2_, T_3_ and T_4_ (*p* > 0.05). The MPV at T_5_ was significantly higher in group C than in group A (*p* < 0.05) (Fig. 2). The ratio of MPV to the platelet (PLT) counts in the three groups increased at T_2_ and reached the peak at T_4_, and then decreased gradually (Fig. 3).

**Figure 4:**
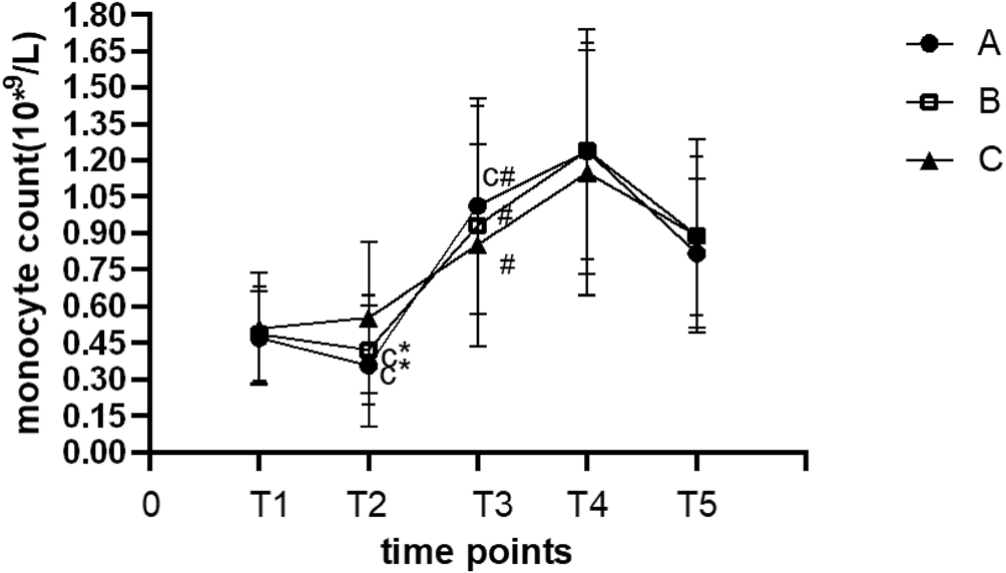
The absolute monocyte count at different time points in all groups. *p < 0.05 vs. T1; ^#^*p* < 0.05vs. T2; ^c^*p* < 0.05 vs. group C.

The absolute monocyte count in groups A and B was significantly lower at T_2_ than at T_1_ (*p* < 0.05). The absolute monocyte count in the three groups was increased at T_3_ and reached the peak at T_4_ (*p* < 0.05), and the absolute monocyte count in group A was lower at T_5_ than at T_3_ (*p* < 0.05). The absolute monocyte count at T_2_ was significantly higher in group C than in groups A and B (*p* < 0.05), while the absolute monocyte count at T_3_ was significantly higher in group A than in group C (*p* < 0.05). (Fig. 4).

**Figure 5:**
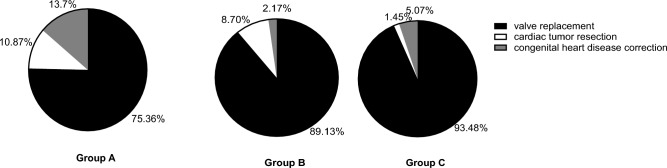
proportion of operation type.

There were no significant differences in age, weight, gender and ASA grade among the three groups (*p* > 0.05) (Table 1). During surgical hypothermia, temperature fluctuated 1.0 °C above and below 30 °C. The valve replacement surgery accounted for the largest proportion of operation types in each group. Proportion of tissue valves in groups A, B and C was 48.7%, 51.2%, 50.4% respectively, and the proportion of mechanical valves in three groups was 51.3%, 48.8%, 49.6% accordingly. Compared with groups B and C, the proportion of congenital heart disease correction and cardiac tumor resection was higher in group A, while the proportion of valve replacement was lower (*p* < 0.05). (Fig. 5).

Compared with groups B and C, the volume of intraoperative blood loss was significantly lower in group A (*p* < 0.05); the volume of plasma infusion was significantly lower in group A than in groups B and C (*p* < 0.05), while there was no difference in the volume of plasma infusion between groups B and C (*p* > 0.05). The dose of heparin, protamine and volume of residual blood were similar among the three groups (*p* > 0.05) (Table 2).

**Table 1:** Characteristics of general materials.

	Group A (n = 140)	Group B (n = 149)	Group C (n = 138)	*F*/*X*^2^ value	*P* value
Age(years)	54.6 ± 11.0	57.2 ± 9.9	56.0 ± 12.1	1.930	0.146
Weight (Kg)	57.1 ± 9.0	57.3 ± 9.2	58.7 ± 10.5	1.182	0.308
Gender (male/female)	62/78	65/84	73/65	3.020	0.221
ASA grade				0.535	0.827
II	5(3.57%)	5(3.36%)	3(2.17%)		
III	135(96.43%)	144(96.64%)	135(97.83%)		
ECC time(min)	36.0–120.0 (97.4 ± 19.7)	121.0–180.0 (149.8 ± 18.0)	181.0–415.0 (223.4 ± 38.1)

**Table 2:** Description of perioperative conditions.

	Group A (n = 140)	Group B (n = 149)	Group C (n = 138)	*F* value	*p* value
Volume of intraoperative blood loss(ml)	357.6 ± 149.2^cb^	386.2 ± 113.5	459.7 ± 375.0	6.533	0.002
Totalheparin dose (IU)	24,500 ± 4850	24,163 ± 3975	25,413 ± 5125	2.626	0.074
Total protamine dose (mg)	285.4 ± 56.0	286.2 ± 60.1	287. 6 ± 57.7	0.050	0.951
Volume ofresidual blood(ml)	372.1 ± 227.4	371.4 ± 250.1	409.4 ± 300.8	0.957	0.385
Volume of perioperative plasma transfusion (ml)	366.1 ± 85.8	388.4 ± 54.6^a^	396.5 ± 41.3^a^	8.531	< 0.001

^a^*p* < 0.05 vs. group A.

^b^*p* < 0.05vs. group B.

^c^*p* < 0.05vs. group C.

In group A, 20 patients (14.29% ) were received intraoperative residual blood transfusion (the lowest infusion volume was 150 ml, the highest infusion volume was 1100 ml), 12 patients (8.57%) were infused with suspended red blood cells (the lowest infusion volume was 1U and the highest infusion volume was 4U); In group B, 29 patients (19.46%) were received intraoperative residual blood infusion (the lowest infusion volume was 200 ml and the highest infusion volume was 800 ml), 22 patients (14.77%) received suspended red blood cells (the lowest infusion volume was 1U and the highest infusion volume was 4U); In group C, 32 patients (23.19%)were received intraoperative residual blood transfusion (the lowest infusion volume was 100 ml, the highest infusion volume was 1350 ml), 32 patients (23.19%) were infused with suspended red blood cells (the lowest infusion volume was 0.7u, the highest infusion volume was 8.5u), one patient (0.73%) was infused with cryoprecipitate10u, three patients (2.17%) were infused with human fibrinogen 1 g, 2 g and 1 g respectively, and three patients (2.17%) were infused with human prothrombin complex 600iu, 1200iu and 600iu respectively. In group C, there were 5 cases of reoperation for hemostasis, and zero cases in the other two groups.

## Discussion

Our study found that platelet count in the three groups after ECC was reduced significantly and negatively correlated with the time of ECC, although the correlation coefficients were low. The MPV dropped significantly at the end of operation in each group, and rebounded rapidly on the first day after operation. The ratio of MPV to the platelet count in the three groups was increased at the end of operation and reached the peak on the second day after operation, and then decreased gradually. The monocyte count was reduced significantly at the end of operation in patients with ECC time less than 3 h, but not in patients with ECC time greater than 3 h. The monocyte count in the three groups was increased rapidly on the first day after operation, peaked on the second day after operation, and then decreased gradually. In addition, patients with ECC time greater than 2 h had a significant increase in volume of blood loss and plasma infusion.

There are two types of thrombocytopenia after cardiac surgery. Type I is biphasic, that is, platelet count drops first, followed by a brief increase, then falls again, and gradually rises within 5 days after operation; Type II showed that platelet count was continuously lower than that before operation within 7–10 days after operation. These two types of thrombocytopenia are common in heparin induced thrombocytopenia (HIT). Such changes in platelet count can increase the specificity of diagnosing HIT^2^. The mechanism leading to the two types of changes is unclear and needs to be further studied. We found that platelet count after ECC decreased significantly compared with that before operation. Platelet count in each group increased slightly on the first day after operation, decreased to the level after operation on the second day, and increased significantly on the fifth day after operation. The change is consistent with the characteristics of type I. Thrombocytopenia often occurs after ECC, and the maximum drop usually occurs between 48 and 72 h after ECC, and the lowest platelet count after ECC can be reduced to half of that before ECC^1^. We found that platelet count decreased significantly after ECC, and decreased to the lowest on the 2nd day after ECC, which is consistent with the above study. It shows that ECC in open heart surgery can significantly reduce the platelet count. Clinically, it is necessary to continuously monitor the platelet count after cardiac surgery under ECC in order to deal with it in time. The significant decrease of platelet count after ECC is caused by hemodilution and mechanical damage. The maximum drop in platelet count on 2–3 days after ECC is mainly due to the reaction between oxygenator membrane and blood, shear force during ECC and the use of heparin, by which platelets are activated and consumed^2^. In our study, platelet count in each group increased significantly on the 5th day after operation. Study^3^ reported that platelet count after ECC recovered within 5–6 days after operation, and there will be no decrease in platelet count after that, which is consistent with our results. It is suggested that platelet count after ECC can be restored in a short time after ECC, and that 5–6 days after operation is a time point to pay attention to platelet count. If platelet count does not rise in time, we need finding the cause and early intervention. The study^4^ considered that platelet count after ECC decreased more seriously with the extension of ECC time. Our study found that platelet count profiles after ECC, on the first day and on the second day after ECC were consistent with the above research results. The extension of ECC time aggravates the mechanical damage to platelets during ECC, and the activated platelets also increase. ECC can also promote the release of platelet factor 4 (PF4). PF4 combines with heparin to form a complex, which can activate and consume platelets, resulting in a significant decrease in platelet count with long ECC time^2^. It is suggested that shortening the ECC time as much as possible in clinical practice can avoid too much destruction of platelets, so as to reduce the degree of decline in platelet count after operation. In our study, platelet count on the 5th day after operation almost returned to the preoperative level when the ECC time was less than 2 h, while ECC time exceeded 2 h, the platelet count remained significantly lower than the preoperative level. The results showed that the extension of ECC time also delayed the recovery of platelet count in patients undergoing cardiac surgery. Thrombopoietin (TPO) is an important factor to platelets production in vivo. After TPO binds to TPO receptor MPL on the cell surface, it activates PI3-K/Akt and Raf-1/Map signaling pathways, promotes the maturation of megakaryocytes, and stimulates the production of platelets. TPO is mainly produced by liver and bone marrow stromal cells. Interleukin-6 (IL-6) can stimulate liver to produce TPO. Platelets derived growth factor and fibroblast growth factor-2 promote bone marrow stromal cells to produce TPO, while PF4, thrombin reactive protein and transforming growth factor- β inhibit TPO production^5^. The study^6^ found that TPO increased gradually after ECC, reached a maximum value 2.5 days after ECC, and the peak value of TPO was consistent with the time point at which platelet count was the lowest, and then decreased to the preoperative level 12 days after ECC. The decrease of platelet mass was two days before the peak of TPO, and the peak of TPO was before the recovery of platelet mass. In addition, the increase of IL-6 was before TPO, and IL-6 reached the highest value two days earlier than TPO. It is suggested that after ECC, the production of TPO can be enhanced by increasing the production of IL-6, and TPO can promote the maturation and division of megakaryocytes to produce platelets, and the peak value of TPO is consistent with the time point at which the platelet count is the lowest. This timely and effective compensation may be one of the reasons for the recovery of the platelet count around 5 days after ECC. The influence and mechanism of different ECC time on platelet count after ECC are not clear and need to be further studied. The decrease in the platelet count after operation is an independent risk factor for massive postoperative bleeding, and patients with reduced platelet count after operation have a higher chance of intraoperative infusion of fresh frozen plasma^7^. Our study found that patients with ECC time longer than 2 h had a significant increase in blood loss and a corresponding increase in plasma infusion. Also, there were 5 cases of reoperation for hemostasis in the group with longer ECC time, which suggested that patients with longer ECC time may increase the risk of bleeding due to severe thrombocytopenia.

The relationship between platelet volume and platelet maturity is controversial. Some people believe that platelets become smaller after maturity and immature platelets are larger. Some people do not support this view, but it is widely believed that platelets with increased volume have stronger hemostatic function reactivity^8^. Immature platelets have a large volume and contain more thrombogenic particles, such as TXA2 (thromboxane A2), TXB2 (thromboxane B2) and GP IIb IIIa receptor (glycoprotein IIb IIIA), so larger platelets are metabolically and enzymatically more active, displaying a greater prothrombotic potential than smaller platelets.^9^. Platelets not only have coagulation function, but also play an important role in immune inflammation. Human complement system mediates the reaction between platelets and leukocytes. Complement can also activate platelets and promote their participation in pro-inflammatory response, so as to exert the immune function of platelets^10^. Study reported that systemic inflammatory response syndrome appeared in the body after ECC^11^. ECC enhanced the immune function of platelets and activated platelets to participate in the body's inflammatory response. The ratio of MPV to PLT increased immediately at the beginning of ECC, continued to increase until it reached the highest value two days after operation, and began to decrease on the fourth day after operation^12^. Our study found that the ratio of MPV to PLT was increased after operation, peaked on the 2nd day after operation, and then decreased gradually. ECC could induce inflammatory response, and the inflammatory reaction reached the strongest on the 2nd day after operation. Attention should be paid to this time point, and anti-inflammatory measures should be taken if necessary. It is reported that the product of MPV and PLT is a constant. When MPV increases, platelet counts decrease^13^. In addition, MPV can reflect the inflammatory response level of the body. When the body is in strong inflammatory response, the MPV level increases, and platelets with high MPV quickly migrate to the inflammatory site to play the immune function, resulting in the consumption of platelets. Therefore, on the 5th day after operation, MPV in the group with ECC time > 3 h is higher than that in the group with ECC time < 2 h, but platelet counts in the group with ECC time > 3 h are lower than that in the group with ECC time < 2 h. We also found that the MPV of the three groups after operation was significantly lower than that before operation, which may be related to the high reactivity of platelets with large MPV, which were cleared by the body after activation, but the body failed to quickly produce immature and large MPV platelets^14^. The specific reason is not clear and needs to be further studied. MPV increased on the first day after operation in the three groups, and the ratio of MPV to PLT reached the peak on the second day after operation, suggesting that it is necessary to take measures to reduce the body’s inflammatory response during and after ECC, in order to reduce the peak value and reduce the activation and destruction of platelets, so as to protect them. The MPV in the group with ECC time > 3 h on the 5th day after operation was significantly higher than that in the group with shorter ECC time, suggesting that in the patients with long ECC time, platelets may play an immune function and participate in the body's stress and immune response, and even this intraoperative response can last until after operation. Shortening the ECC time as far as possible can reduce the consumption of platelets participating in immunity and protect platelets. The specific mechanism needs to be further studied.

Monocytes are one of the cells involved in inflammation, playing a role in phagocytosis, antigen presentation and cytokine production in immune response. After leaving the bone marrow that produces monocytes, monocytes stay in the blood circulation for 1–3 days, and then differentiate into macrophages and dendritic cells^15^. Monocytes were related to inflammation related complications after ECC^16^. After ECC, patients’ blood volume were diluted, and the production of monocytes would take a certain time. Therefore, the monocyte count in the group with ECC time less than 3 h is lower than that before ECC. ECC can lead to systemic inflammatory response. After operation, the body is in inflammatory stress^11^. Monocytes participate in the inflammatory response and continue to compensatory proliferation. A large number of monocytes were activated 24 h after ECC, and monocytes count increased^16^. In our study, the monocyte count increased on the first day after operation, peaked on the second day after operation, and then decreased gradually. It is suggested that the second day after ECC was the time when the body's inflammatory response was the strongest, and we can take measures to control the inflammation and minimize the damage of the body’s inflammatory response to platelets and other related adverse consequences properly. Monocytes can be divided into classical type (CD14++CD16−), non -classical type (CD14+CD16++) and intermediate type (CD14++CD16+). Classical monocytes account for a large proportion and mainly play the role of scavengers. Non -classical monocytes are usually called proinflammatory cells, which are secondary to the mobilization of diseases and secretion of important inflammatory cytokines (such as TNF- α). Disease status can affect the production of monocytes and the conversion between different types. At present, it is generally believed that classical monocytes can produce non-classical and intermediate monocytes^17^. In group C, which the ECC time was longer, the blood was in contact with the oxygenator membrane, and a large number of inflammatory factors were produced due to long-term stress and inflammatory reaction, which makes the classical monocytes produce non-classical cells. Therefore, the monocyte count in group A and group B are significantly lower than that in group C. The specific mechanism needs further phenotypic identification of monocytes. The plasma β 2 micro-globulin (beta-2 micro-globulin, β 2 M) was mainly derived from platelets, and β 2 M can regulate the polarization of monocytes^18^. Activated platelets exert a direct pro-inflammatory effect on monocytes by expressing leukocyte adhesion molecules and releasing a variety of immunomodulatory factors. In addition, activated platelets showed high affinity for monocytes and formed monocyte-platelet complexes under the action of a variety of cell adhesion factors produced by both platelets and monocytes^19^. We speculated that the monocyte count in group C is significantly lower than that in group A on the first day after operation was due to the longer ECC time in group C, which led to a strong inflammatory reaction, and monocytes are consumed to participate in the inflammatory reaction. On the other hand, it may be that a large number of activated platelets formed complexes with monocytes, and macrophages and dendritic cells played the role of phagocytosis and antigen presentation, consuming themselves, and feedback promoting monocyte differentiation, as a result, the monocytes count in group C was significantly lower than that in group A on the first day after operation, the specific mechanism needs to be further studied.

The proportion of operation types was different in the three groups, but valve replacement was the main operation in each group. Patients who underwent congenital heart disease correction and cardiac tumor resection were more in group A than in groups B and C, while patients who underwent valve replacement was fewer in group A. The decrease of PLT counts in biological valve replacement patients was more severe than that in patients with mechanical valve replacement^20^, which was consistent with our result. However, the thrombocytopenia caused by biological valve replacement does not appear to have any effect on postoperative complications, and the PLT counts can recover within one week without any intervention. Glutaraldehyde treatment during the construction of bioprosthetic valves maybe the cause of thrombocytopenia observed after bioprosthetic heart valve replacement^20^. The specific reason is not clear, which needs to be further studied.

Research^21^ showed that infusion of suspended red blood cells can lead to increased platelet activation in patients. It is considered that infusion of red blood cells may directly affect PLT reactivity in ADP pathway by activating P2Y12 platelet receptor or through agonists or mediators contained in red blood cell package, and the specific mechanism was not clear. We observed that with the extension of ECC time, more patients needed infusion of suspended red blood cells. Autologous blood reinfusion can significantly reduce the infusion of suspended red blood cells, platelets, fresh frozen plasma and other blood products in ECC patients from intraoperative to 12 h after operation, playing a role in blood protection and indirectly protect platelets^22^. In our study, patients with ECC time greater than 2 h had a significant increase in volume of blood loss and autologous blood infusion. The existing data of our study were limited and the PLT function was not monitored, but the most types and quantities of blood products infused appeared in the group with ECC time longer than 3 h. It indicated that the coagulation function was seriously damaged with the extension of ECC time. This also reminds us of the need for routine monitoring of intraoperative and postoperative coagulation and platelets function in patients undergoing ECC in the future.

## Limitations

This study is retrospective, due to the limited previous data, it has the following limitations. Firstly, previous data did not monitor the postoperative platelet function continuously, so the related effects of different ECC time on platelet function were unclear. Secondly, the effects of anesthetics on the number and function of platelet were not discussed in this experiment. Thirdly, the influence of ECC on the factors promoting platelets production and the specific mechanism of platelets exerting immune function and participating in immune response during ECC were unclear, which needs to be further studied. Fourthly, it is unclear whether different types of surgery affect the number and function of platelets, which needs to be studied in the future. Fifthly, due to the limited data, the MPV and monocyte count did not return to the preoperative level. Sixthly, our findings may not be generalizable, and more randomized control trials investigating the mechanism of ECC with different time on platelet count and function may be required to confirm our results. In the future, we will conduct multicenter randomized controlled trials to verify our conclusions.

## Conclusion

Thrombocytopenia was common after ECC, maximum drop of the platelet counts after ECC was usually seen on the second day after ECC, and platelet counts started to recover on the fifth day after ECC. With the extension of ECC time, the drop in platelet counts is more pronounced, the volume of perioperative blood loss and blood products transfusion are more, and the recovery level and speed of platelet counts is lower.

## Data Availability

For reasonable data request, contact the corresponding author by email.
